# IMproving PULmonary hypertension Screening by Echocardiography: IMPULSE

**DOI:** 10.1186/s44156-022-00010-9

**Published:** 2022-10-19

**Authors:** Oliver Graham Slegg, James Alexander Willis, Fiona Wilkinson, Joseph Sparey, Christopher Basil Wild, Jennifer Rossdale, Robert Mackenzie Ross, John D. Pauling, Kevin Carson, Sri Raveen Kandan, David Oxborough, Daniel Knight, Oliver James Peacock, Jay Suntharalingam, John Gerard Coghlan, Daniel Xavier Augustine

**Affiliations:** 1 Royal United Hospitals Bath NHS Foundation Trust, Bath, BA13NG UK; 2 School of Healthcare Science, Manchester Metropolitan University, Manchester, UK; 3 Liverpool John Moores University, Liverpool, UK; 4 Royal Free Hospital, London, UK; 5 Department for Health, University of Bath, Bath, UK

**Keywords:** Echocardiography, Pulmonary hypertension, RV free wall longitudinal strain

## Abstract

**Background:**

The world symposium on pulmonary hypertension (PH) has proposed that PH be defined as a mean pulmonary artery pressure (mPAP) > 20 mmHg as assessed by right heart catheterisation (RHC). Transthoracic echocardiography (TTE) is an established screening tool used for suspected PH. International guidelines recommend a multi-parameter assessment of the TTE PH probability although effectiveness has not been established using real world data.

**Study aims:**

To determine accuracy of the European Society of Cardiology (ESC) and British Society of Echocardiography (BSE) TTE probability algorithm in detecting PH in patients attending a UK PH centre. To identify echocardiographic markers and revised algorithms to improve the detection of PH in those with low/intermediate BSE/ESC TTE PH probability.

**Methods:**

TTE followed by RHC (within 4 months after) was undertaken in patients for suspected but previously unconfirmed PH. BSE/ESC PH TTE probabilities were calculated alongside additional markers of right ventricular (RV) longitudinal and radial function, and RV diastolic function. A refined IMPULSE algorithm was devised and evaluated in patients with low and/or intermediate ESC/BSE TTE PH probability.

**Results:**

Of 310 patients assessed, 236 (76%) had RHC-confirmed PH (average mPAP 42.8 ± 11.7). Sensitivity and specificity for detecting PH using the BSE/ESC recommendations was 89% and 68%, respectively. 36% of those with low BSE/ESC TTE probability had RHC-confirmed PH and BSE/ESC PH probability parameters did not differ amongst those with and without PH in the low probability group. Conversely, RV free wall longitudinal strain (RVFWLS) was lower in patients with vs. without PH in low BSE/ESC probability group (− 20.6 ± 4.1% vs − 23.8 ± 3.9%) (*P* < 0.02). Incorporating RVFWLS and TTE features of RV radial and diastolic function (RVFAC and IVRT) within the IMPULSE algorithm reduced false negatives in patients with low BSE/ESC PH probability by 29%. The IMPULSE algorithm had excellent specificity and positive predictive value in those with low (93%/80%, respectively) or intermediate (82%/86%, respectively) PH probability.

**Conclusion:**

Existing TTE PH probability guidelines lack sensitivity to detect patients with milder haemodynamic forms of PH. Combining additional TTE makers assessing RV radial, longitudinal and diastolic function enhance identification of milder forms of PH, particularly in those who have a low BSE/ESC TTE PH probability.

**Supplementary Information:**

The online version contains supplementary material available at 10.1186/s44156-022-00010-9.

## Introduction

Pulmonary hypertension (PH) is a relatively common complication of lung or heart disease and more rarely from pulmonary vascular abnormalities for example proliferative vasculopathy or thromboembolic obstruction. Irrespective of cause, PH is associated with increased mortality and morbidity [1]. Historically, PH has been defined as a mean pulmonary artery pressure (mPAP) of ≥ 25 mmHg at rest as assessed by right heart catheterization (RHC) [2–4]. At the World symposium on Pulmonary Hypertension in 2018 a proposal was made to lower the diagnostic threshold to a mPAP > 20 mmHg as this represents 2SD above normal [3].

Untreated PH leads to right ventricular (RV) dysfunction and ultimately failure resulting in exertional dyspnoea, presyncope, chest pain and peripheral oedema. Early identification of PH allows prompt intervention with increasingly effective therapeutic interventions. However, many years may pass between the onset of symptoms and correct diagnosis, delaying potential treatment [5, 6].

Transthoracic echocardiography (TTE) is an established screening tool to asses non-invasively for markers of PH. TTE is a key screening tool due to its wide availability, portability, and cost effectiveness [4, 7, 8].

Doppler TTE estimates of PASP are frequently discordant with RHC evaluation [9–11]. Doppler TTE PASP estimates show good correlations across patient populations but with only moderate precision on an individual basis. Such imprecision can lead to both under and overestimation of true PASP resulting in either a failure to identify PH or lead to unnecessary invasive diagnostic tests [4, 12].

**Fig. 1:**
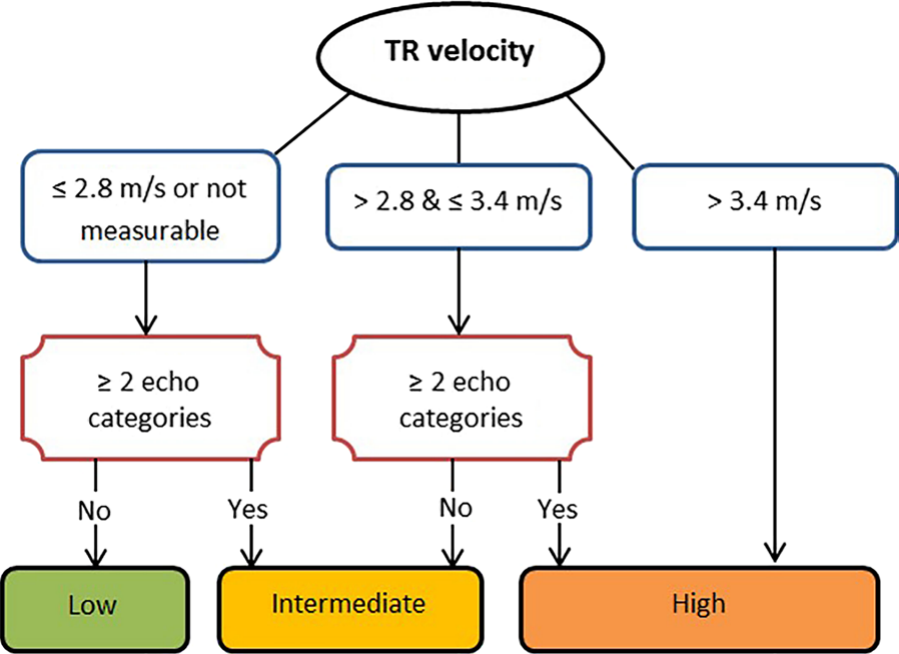
Flow chart to assess the probability of pulmonary hypertension using parameters identified from within ≥ 2 categories (the ventricles, pulmonary artery or the inferior vena cava and right atrium) in conjunction with tricuspid regurgitation velocity. Adapted from [3]

For this reason, both European Society of Cardiology (ESC) [2] and British Society of Echocardiography (BSE) [4] recommend a multi parameter assessment of the TTE probability of PH that incorporates assessment of the peak tricuspid regurgitation velocity (TRV) together with three main categories (A: The ventricles B: Pulmonary artery C: Inferior vena cava (IVC) and right atrium). These measures help to evaluate RV size and pressure overload, the profile of blood flow velocity at the pulmonary valve, the diameter of the pulmonary artery (PA) and an estimate of right atrial pressure [2]. When determining the BSE/ESC TTE probability of PH, the TRV is used in conjunction with the TTE markers described in Table 1 to assign the probability of PH being present (see Fig. 1) [4]. Following ESC and BSE guideline implementation [2, 4] there has been limited research in real world populations to assess the effectiveness of these recommendations in detecting PH.

**Table 1:** Echocardiographic parameters used to assess the echocardiographic probability of pulmonary hypertension

A: The ventricles*	B: Pulmonary artery*	C: Inferior vena cava and right atrium*
Right ventricle/left ventricle basal diameter ratio > 1.0	Right ventricular outflow Doppler acceleration time < 105 ms and/or mid systolic notching	Inferior vena cava diameter > 21 mm with decreased inspiratory collapse (< 50% with a sniff or < 20% with quiet respiration)
Flattening of the interventricular septum (left ventricular eccentricity index > 1.1 in systole or both systole and diastole)	Early diastolic pulmonary regurgitation velocity > 2.2 m/s	Right atrial area (end systole) > 18 cm^2^
	Pulmonary artery diameter > 25 mm	

Adapted from British Society of Echocardiography guidelines on the assessment of suspected pulmonary hypertension [4]. *Echocardiographic parameters from at least two different categories (A/B/C) from the list should be present to alter the level of echocardiographic probability of pulmonary hypertension

Research has demonstrated that right ventricular free wall longitudinal strain (RVFWLS) is reduced in those with PH compared to controls [13, 14]. Evidence also suggests that RVFWLS may provide important prognostic information in those with PH [13, 14]. It has also been hypothesised that in those with PH a reduction in radial RV function is one of the first parameters to deteriorate, therefore assessment by right ventricular fractional area of change (RVFAC) may be a particularly sensitive indicator of elevated pressures [15]. Furthermore, markers of RV diastolic dysfunction are abnormal in those with PH compared to controls [16]. However, to our knowledge no previous studies have looked at the added predictive value of markers including RVFWLS, RVFAC and RV diastolic function indices in the TTE screening of those with suspected PH.

The aims of this study were to determine the accuracy of the BSE/ESC TTE probability algorithm in detecting PH in a real-world population being assessed in a shared care UK PH centre. The study population was used as a derivation cohort to identify other echocardiographic markers which may help to improve identification of those with PH and either low or intermediate BSE/ESC TTE PH probability.

## Methods

### Subjects and study procedures

Patients attending for initial evaluation of PH between August 2010 and March 2020 were assessed. Exclusion criteria included those with known PH; RHC not performed within 4-months of TTE or RHC performed prior to TTE. A clinical diagnosis of pre-capillary PH was defined as a RHC mPAP > 20 mmHg and PVR ≥ 3 woods units (WU). Post-capillary PH was defined as a mPAP > 20 mmHg and a PCWP > 15 mmHg, as per ESC and WSPH guidelines [3]. PH aetiologies were categorised according to the WHO classifications of PH [3].

RHC was only performed in those with a strong clinical suspicion of PH. RHC in those with low TTE PH probability was only performed in those with symptoms consistent with suspected PH, strong risk factors or suspicion of PH from other imaging modalities.

Relevant demographic and clinical characteristics were recorded. Any echocardiographic parameters required to calculate the BSE/ESC TTE PH probability that had not been reported at the time of the initial test were retrospectively re-measured by two experienced echocardiographers blinded to PH status. PH TTE probabilities were calculated following BSE/ESC [2, 4] guidance.

Echocardiographic measures of right heart size and function were performed in accordance with BSE guidelines [17]. LV dedicated AFI speckle-tracking software was used to measure RVFWLS. An average of the basal, mid and apical RV free wall segments was used to quantify RVFWLS. For the purposes of this study more positive RVFWLS values are referred to as ‘lower RVFWLS’ to emphasise abnormality.

Ethics approval was obtained from both the RUH Bath NHS Foundation Trust R&D department and Manchester Metropolitan University Ethos ethics committee.

### Development of IMPULSE algorithm

**Fig. 2:**
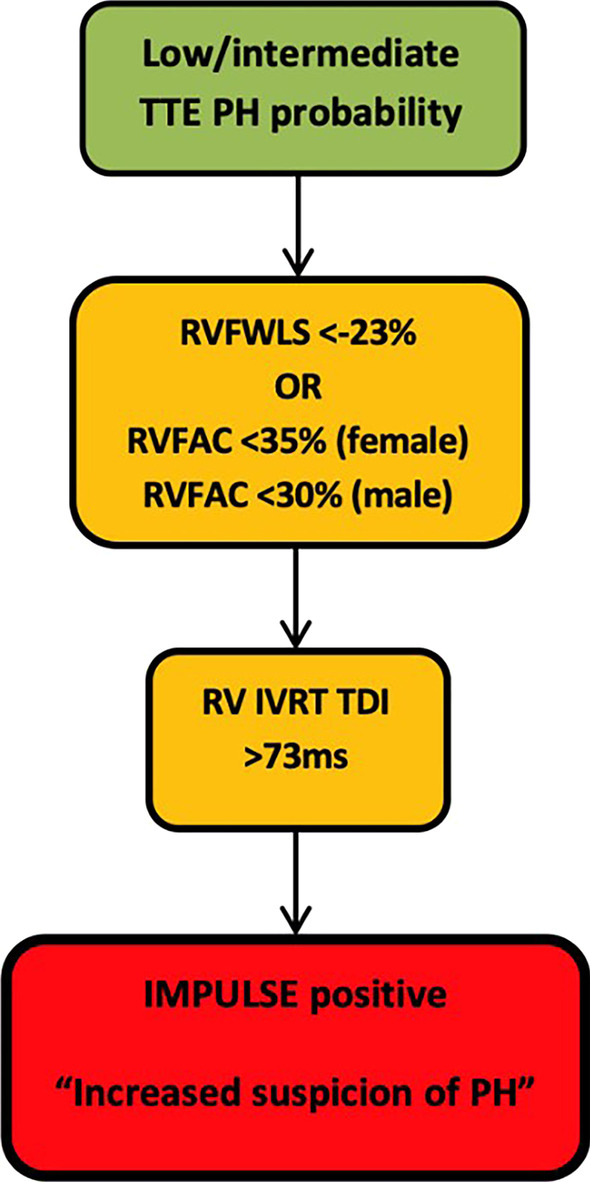
Proposed additional algorithm for those with low or intermediate TTE probability to help identify those with PH and low TTE probability. RVFAC normal cut-off values taken from (Harkness et al., 2020). RVFWLS (right ventricular free wall longitudinal strain); RVFAC (right ventricular fractional area change); IVRT TDI (isovolumetric relaxation time by tissue Doppler imaging); IMPULSE (improving pulmonary hypertension screening by echocardiography)

The IMPULSE algorithm (Fig. 2) was developed to see whether including additional markers of RV longitudinal, radial and diastolic function could help identify PH in those with a low or intermediate TTE probability. Markers including RVFWLS, RVFAC and RV TDI IVRT were chosen as they could be measured retrospectively and had acceptable diagnostic accuracy when evaluated by ROC analysis.

### Statistical analysis

All statistical analysis was performed using SPSS version-26. Categorical variables are summarised as percentage frequencies and continuous variables as mean ± SD. The dataset was tested for normality using the Shapiro–Wilk test. Proportions were compared using the Chi-squared test and Fisher’s exact test. Comparisons of means were analysed using the Mann–Whitney U tests and Kruskal–Wallis tests. Receiver operating characteristic (ROC) curve analysis was used to establish optimal sensitivities and specificities for the individual PH TTE parameters.

When calculating sensitivities and specificities of the ESC/BSE TTE PH probability algorithm those with either high or intermediate TTE probability were deemed as positives for PH whilst those with low TTE PH probability were deemed as negative for PH.

Univariate Cox proportional hazard ratios were determined to assess the predictive value of RVFWLS for all-cause mortality in PH. Kaplan–Meier survival curve analysis was used to estimate the distribution of time from PH diagnosis to all-cause mortality. ROC analysis was used to determine the optimal cut-off RVFWLS value for predicting all-cause mortality.

## Results

### Clinical and functional characteristics

Of 498 patients undergoing TTE assessment, 310 eligible patients were identified for this analysis. Their clinical characteristics are described in Additional file 1: Table S1. There was a female predominance (62%) with a mean age of 67 ± 14 years. The median time from TTE to RHC was 24 days (IQR, 8–46 days).

A summary of clinical and haemodynamic parameters in those with PH according to BSE/ESC TTE PH probability is shown in Table 2. Seventy-six percent of the cohort (N = 236) had RHC PH (average mPAP of 42.8 ± 11.7 mmHg) with the most frequent aetiology being chronic thromboembolic PH (CTEPH). Average NT-proBNP levels were higher in those with PH (1817.6 ± 2123 ng/L) when compared to those without (286 ± 459.8 ng/L) (P < 0.001). Those with PH covered significantly lower six-minute walk test (6MWT) distance (299.9 ± 153.2 m) compared to those without PH (372.9 ± 127.2 m, P < 0.001).

**Table 2:** Clinical and haemodynamic parameters in those with either low, intermediate or high BSE/ESC TTE PH probability (N = 310)

TTE parameters	Low (N = 78)	Intermediate (N = 68)	High (N = 164)
PAH	4	17	42
LHD	19	14	28
CTEPH	5	11	64
Lung disease		2	6
Multifactorial/unclear		6	18
No PH	50	18	6
Haemodynamic data	(N = 78)	(N = 68)	(N = 164)
mPAP (mmHg)	31.3 ± 6.9	37.4 ± 9.7	46.3 ± 11.2
PVR (mmHg)	3.0 ± 1.6	5.4 ± 3.2	7.7 ± 3.8
PCWP (mmHg)	15.1 ± 4.8	14.1 ± 5.4	16.9 ± 6.1
PA sats (%)	71.6 ± 4.0	66.1 ± 8.2	63.0 ± 8.7
CO (L/min)	5.1 ± 1.4	4.7 ± 1.3	4.3 ± 1.2
CI ((L/min/m^2^)	2.8 ± 0.7	2.5 ± 0.7	2.4 ± 0.6
NT Pro BNP (ng/L)	321.1 ± 341.6	1039.7 ± 1393.9	2282.5 ± 2995.5
6MWT (m)	318.9 ± 178.7	298.6 ± 164.4	262.7 ± 144.7

*PAH* pulmonary arterial hypertension, *LHD* left heart disease, *CTEPH* chronic thromboembolic pulmonary hypertension, *mPAP* mean pulmonary artery pressure, *PVR* pulmonary vascular resistance, *PCWP* pulmonary capillary wedge pressure, *PA sats* pulmonary artery saturations, *CO* cardiac output, *CI* cardiac index, *NT pro BNP* N-terminal pro hormone of brain natriuretic peptide, *6MWT* six-min walk test

### ESC/BSE TTE probability of PH

**Fig. 3:**
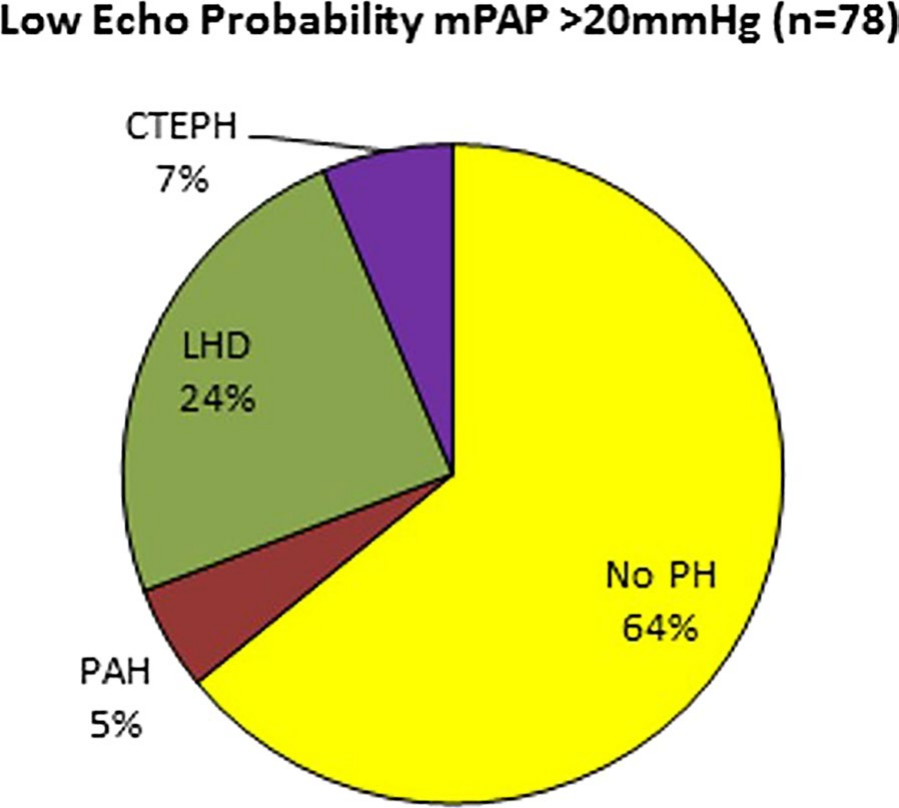
Comparison of the frequency of WHO Classification of PH in those with an low echocardiographic probability of PH (N = 78)

TTE PH probabilities (high N = 164; 53%, intermediate N = 68; 22% and low 78; 25%) and aetiologies of PH are further detailed in Table 2. Ninety-six percent and 75%, of those with high or intermediate TTE probability had RHC PH, respectively. Thirty-six percent (N = 28) of those with low TTE probability had PH definitively diagnosed at RHC. Their PH aetiologies are depicted in Fig. 3. The four patients with PAH all had systemic scleroderma which accounted for 9% of all those in the cohort with systemic scleroderma associated PH. The calculated sensitivity and specificity of the ESC/BSE TTE algorithm for detecting PH was 88% and 68%, respectively.

The peak TRV was measurable in 83% of the overall cohort and in 92% of those with RHC PH. However, the peak TRV was only measurable in 63% of those with a low TTE probability of PH. Most other conventional TTE parameters were frequently measurable (Table 3). However, the main pulmonary artery (MPA) diameter and pulmonary regurgitation velocity at beginning of diastole (PRV_BD_) were frequently unmeasurable (measurements able to be made in 40% and 30%, respectively).

**Table 3:** Comparison of TTE PH parameters in those with and without PH

TTE parameters	PH (N = 236)	No PH (N = 74)	Statistical significance
Peak TRV (m/s) (N = 257)	3.6 ± 0.84	2.56 ± 0.4	P < 0.001
RV/LV BASAL diameter ratio (N = 249)	1.01 ± 0.31	0.78 ± 0.21	P < 0.001
End systolic eccentricity index (N = 274)	1.28 ± 0.36	0.99 ± 0.18	P < 0.001
End diastolic eccentricity index (N = 267)	1.12 ± 0.28	0.90 ± 0.17	P < 0.001
RVOT acceleration time (ms) (N = 290)	81 ± 25	109 ± 31	P < 0.001
MPA diameter (mm) (N = 125)	24 ± 6	20 ± 5	P < 0.01
PRV_BD_ (m/s) (N = 92)	2.34 ± 0.41	1.5 ± 0.36	P < 0.001
Right atrial area (cm^2^) (N = 310)	20.9 ± 8.4	15.4 ± 5.8	P < 0.001
Frequency with IVC diameter > 21 mm and reduced collapsibility (N = 273)	N = 41 (17%)	N = 2 (3%)	P < 0.001
RVFWLS (-%) (N = 155)	− 17.4 ± 6.8	− 24.1 ± 5.1	P < 0.001
RVFAC (%) (N = 81)	27.2 ± 12	39.1 ± 9.7	P < 0.001
Indexed right atrial area (cm^2^/m^2^) (N = 268)	11.16 ± 4.6	8 ± 3.4	P < 0.001
RV TDI E’ (N = 228)	9.02 ± 3.45	10.63 ± 3.19	P < 0.01
RV TDI E’/A’ ratio (N = 219)	0.71 ± 0.52	0.82 ± 0.36	P < 0.01
RV TDI IVRT (N = 164)	98.1 ± 39.2	76.5 ± 26.2	P < 0.001

*TTE* transthoracic echocardiography, *TRV* tricuspid regurgitant velocity, *RV* right ventricle, *LV* left ventricle, *PRV*_*BD*_ pulmonary regurgitant velocity at begging of diastole, *MPA* main pulmonary artery, *RVOT* right ventricular outflow tract, *MSN* mid-systolic notching, *IVC* inferior vena cava, *RVFWLS* right ventricular free wall longitudinal strain, *TDI* tissue doppler imaging, *IVRT* isovolumetric relaxation time, *RVFAC* right ventricular fractional area of change

### Accuracy of echocardiographic parameters in those with PH

A comparison of TTE markers in those with and without PH can be seen in Table 3. Table 4 demonstrates ROC analysis of the TTE PH parameters. The peak TRV displayed the greatest diagnostic accuracy. The PRV_BD_ similarly demonstrated good diagnostic accuracy with very high specificity but low sensitivity.

**Table 4:** Receiver operating characteristic analysis of the TTE PH parameters

TTE parameter	BSE Cut-off value	AUC	95% CI	P-value	Sensitivity (%)	Specificity (%)
Lower peak TRV (m/s) (N = 257)	> 2.8	0.87	0.83–0.91	P < 0.001	83	73
Upper peak TRV (m/s) (N = 257)	> 3.4	0.87	0.83–0.91	P < 0.001	60	98
End systolic eccentricity index (N = 274)	> 1.1	0.78	0.72–0.84	P < 0.001	64	83
End diastolic eccentricity index (N = 267)	> 1.1	0.75	0.69–0.81	P < 0.001	46	93
RV/LV basal diameter ratio (N = 249)	> 1.0	0.74	0.67–0.81	P < 0.001	47	91
RVOT acceleration time (ms) (N = 290)	< 105	0.78	0.71–0.84	P < 0.001	84	54
MPA diameter (mm) (N = 125)	> 25	0.71	0.60–0.81	P < 0.01	40	91
PRV_BD_ (m/s) (N = 92)	> 2.2	0.82	0.73– 0.91	P < 0.001	61	94
Right atrial area (cm^2^) (N = 310)	> 18	0.73	0.66–0.79	P < 0.001	57	81
RVFWLS (-%) (N = 155)	< − 23	0.78	0.71–0.85	P < 0.001	81	58
RVFAC (%) (male) (N = 33)	< 30	0.72	0.61–0.83	P < 0.001	65	69
RVFAC (%) (female) (N = 48)	< 35	0.72	0.61–0.83	P < 0.001	64	70
Indexed right atrial area (cm^2^/m^2^) (N = 268)	> 11	0.74	0.67–0.81	P < 0.001	42	89
RV TDI E’ (N = 228)	< 8.5	0.67	0.60–0.74	P < 0.001	55	74
RV TDI E’/A’ ratio (N = 219)	< 0.65	0.66	0.58–0.73	P < 0.001	86	28
RV TDI IVRT (N = 164)	> 73	0.72	0.62–0.82	P < 0.001	81	62

*TTE* Transthoracic echocardiography, *AUC* area under curve, *CI* confidence interval, *BSE* British Society of Echocardiography, *TRV* tricuspid regurgitant velocity, *RV* right ventricle, *LV* left ventricle, *PRV*_*BD*_ pulmonary regurgitant velocity at begging of diastole, *MPA* main pulmonary artery, *RVOT* right ventricular outflow tract, *MSN* mid-systolic notching, *IVC* inferior vena cava, *RVFWLS* right ventricular free wall longitudinal strain, *TDI* tissue doppler imaging, *IVRT* isovolumetric relaxation time, *RVFAC* right ventricular fractional area of change, *RVOT* right ventricular outflow tract, *PRV*_*BD*_ pulmonary regurgitation velocity beginning of diastole, *RVFWLS* right ventricular free wall longitudinal strain, *RVFAC* right ventricular fractional area of change

Other conventional TTE parameters used to assess BSE/ESC PH probability exhibited acceptable diagnostic accuracy typically demonstrating low sensitivity but high specificity for detecting PH (Table 4). A re-audit of the TTE PH probabilities using ROC defined cut-off values did not significantly increase sensitivity or specificity of the BSE/ESC TTE PH probability algorithm (Additional file 1: Tables S2, S3).

### RVFWLS analysis

RVFWLS was measurable in 50% (n = 155) and was significantly lower in those with PH (− 17.4 ± 6.8%) compared to those without (− 24.1 ± 5.1%), (P < 0.001), (Additional file 1: Fig. S1a). RVFWLS was also lower in those with a high TTE PH probability compared to both intermediate and low probability groups (P < 0.001), (Additional file 1: Fig. S1b). RVFWLS demonstrated acceptable diagnostic accuracy with sensitivity and specificity of 81% and 58%, respectively, using a cut-off value of < − 23%. Subgroup analysis in those with pre-capillary PH exhibited sensitivity and specificity of 84% and 60%, respectively.

Interestingly, RVFWLS was significantly lower in those with low TTE probability and PH (− 20.6 ± 4.1%) compared to those without PH (− 23.8 ± 3.9%), (P < 0.02), (Additional file 1: Fig. S2a, b). RVFWLS was also lower in those with intermediate TTE probability and PH (− 20.14 ± 7.48%) compared to those without (− 25 ± 7.34%) (p = 0.09). RVFWLS was only measurable in 1 patient with high TTE probability and no PH with an RVFWLS value of − 23.7%.

**Fig. 4:**
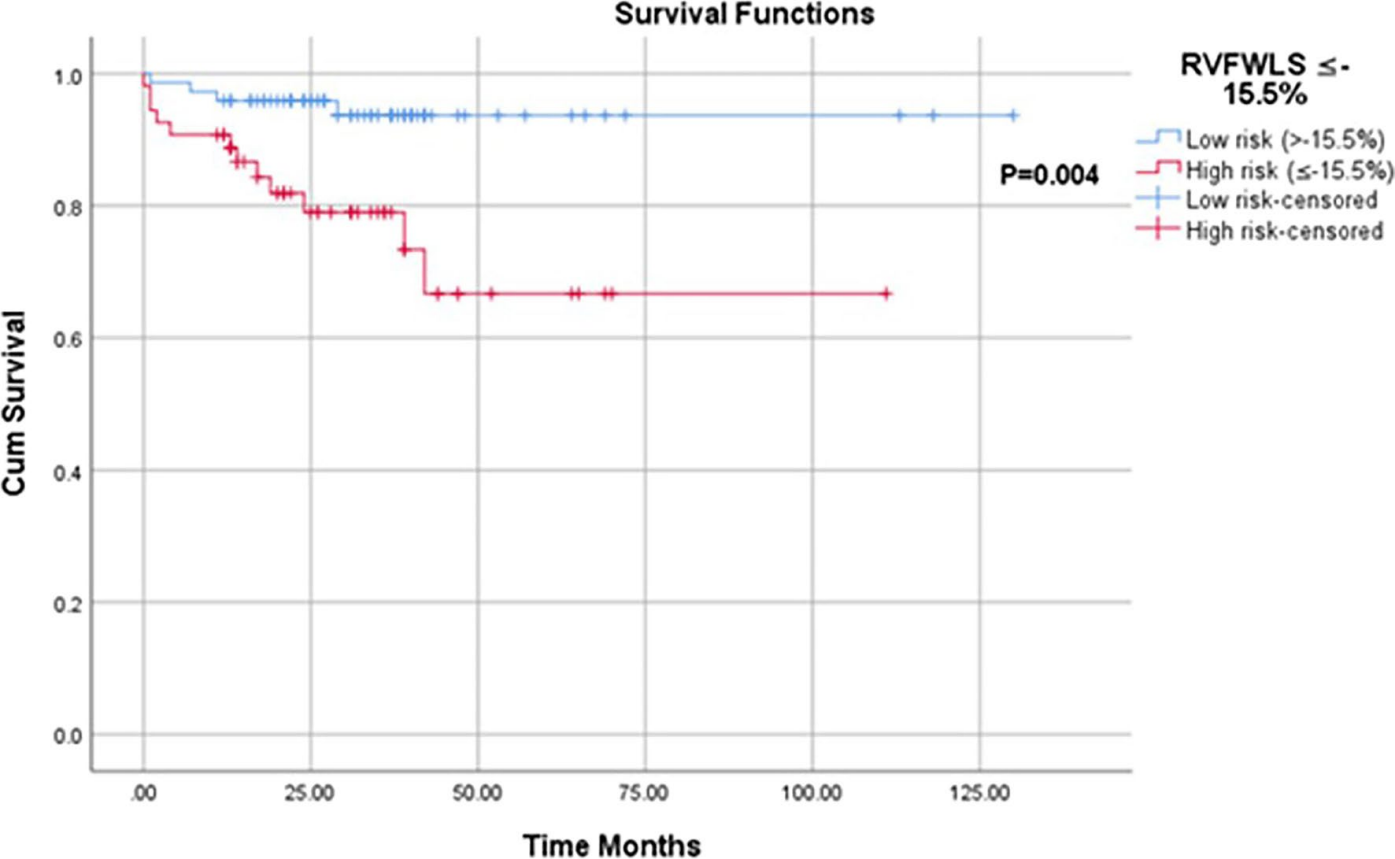
Kaplan–Meier survival curve analysis in 127 PH patients for RVFWLS over a follow-up period of 29 [19–40] months. RVFWLS was categorised into low risk (blue line, n = 73) and high risk (red line, n = 54). ROC derived cut-off RVFWLS value was used to categorise those at low (> − 15%) or high (≤ − 15.5%) risk of all-cause mortality. Kaplan–Meier survival curve analysis demonstrated a significant difference in survival between those with low risk RVFWLS scores and high risk RVFWLS scores (P < 0.01)

There were 38 documented deaths (all-causes) during a median follow-up of 2 years (IQR 1–3 years). Of these deaths, 37 occurred in those with PH and of these 84% (N = 31) had a high TTE PH probability. RVFWLS was significantly lower in those with PH that died (− 14 ± 8% vs − 17.8 ± 6.6%, P < 0.05). Univariate hazard ratios demonstrated an increased risk of all-cause mortality in those with an RVWLS ≤ − 15.5% [4.517, 95% CI 1.454–14.038]. Kaplan–Meier survival analysis demonstrated those with PH and an RVFWLS ≤ − 15.5% had an increased risk of all-cause mortality (p < 0.01) (Fig. 4).

### RV diastolic parameters

Markers of RV diastolic function including indexed RAA, TDI E’, TDI E’:A’ and IVRT by TDI were significantly worse in those with PH compared to those without (all, P < 0.05) (Table 3; Additional file 1: Table S4). A meaningful assessment of RV E:A ratios, deceleration times and E/e’ was not possible as trans-tricuspid PW Doppler was only used in 8% (N = 25).

Of the RV diastolic parameters studied, the TDI IVRT demonstrated the highest diagnostic accuracy when using a cut-off value of > 73 ms (Table 4; Additional file 1: Fig. S3). RV diastolic parameters are further described in Additional file 1: Fig. S4a–c. There was a trend for higher average IVRT values in those with intermediate and low TTE probability and PH compared to those without PH (Additional file 1: Fig. S5a, b).

### RVFAC analysis

RVFAC was significantly lower in those with PH compared to those without (P < 0.001). RVFAC also demonstrated acceptable diagnostic accuracy using a cut-off RVFAC of < 30% in males and < 35% in females. RVFAC was retrospectively measurable in 45% of those with low and intermediate TTE PH probability. In subgroup analysis of those with low TTE probability, RVFAC trended to be lower in those with PH compared to those without (32.7 ± 11.1% vs 38.6 ± 9.7%, P = 0.05) and was significantly lower in those with pre-capillary PH (25.9 ± 8.7% vs 38.6 ± 9.7%, P < 0.01). In those with intermediate TTE probability RVFAC was significantly lower in those with PH compared to those without (27.46 ± 13.1% vs 42.33 ± 10%, P < 0.02).

### Low TTE probability subgroup analysis

**Fig. 5:**
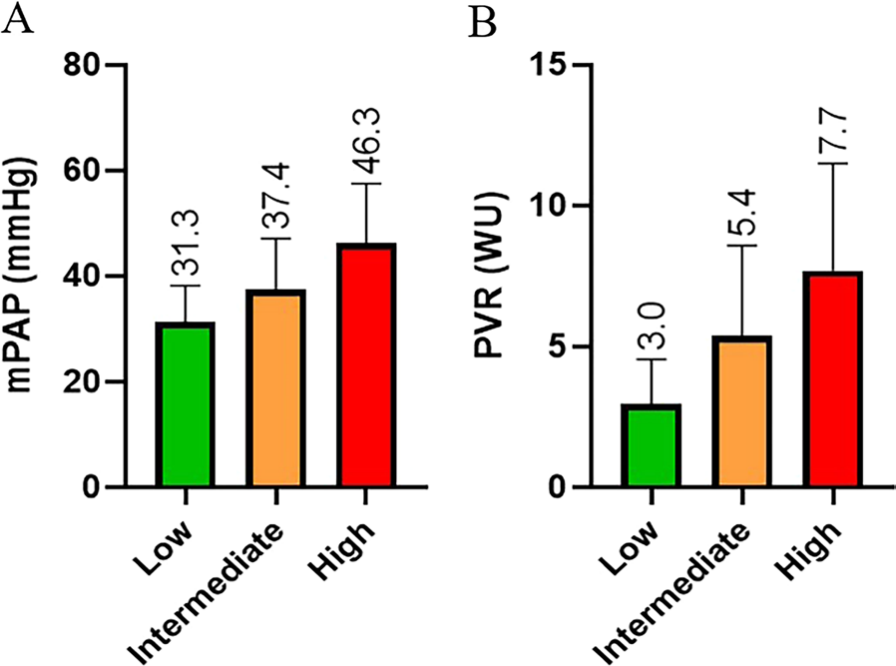
**a** Comparison of mPAP (mmHg) relative to TTE probability of PH. Kruskal–Wallis analysis demonstrated mPAP values were significantly different across TTE PH probability groups; Kruskal–Wallis H = 138.2 (2) (P < 0.0001). Pairwise analysis with adjusted P-values demonstrated mPAP was significantly higher in those with either high or intermediate TTE probability compared to low (both P < 0.0001). mPAP was also significantly higher in those with intermediate compared to low TTE probability (P < 0.0001). mPAP (mean pulmonary artery pressure). **b** Comparison of PVR (woods units) relative to TTE probability of PH. Kruskal–Wallis analysis demonstrated PVR was significantly different across TTE PH probability groups; H = 138.3(2) (P < 0.0001). Pairwise analysis with adjusted P-values demonstrated PVR was significantly higher in those with high or intermediate TTE probability compared to low probability (both P < 0.0001). PVR was also significantly elevated in those with intermediate compared to low TTE probability (P < 0.0001). PVR (pulmonary vascular resistance)

Of those with low TTE probability and PH, mPAP and PVR were significantly lower compared to those with intermediate or high TTE probability (Fig. 5a, b). In those with low TTE probability, there was no significant difference in 6MWT distances, BORG scores or NYHA classifications between those with PH and those without. NT-proBNP values were significantly higher in those with LHD PH compared to those without (462.6 ± 428.1 ng/L vs 148.4 ± 107.7 ng/L, P < 0.01) but not significantly different between those with pre-capillary PH and no PH (152.8 ± 113.2 vs 169.1 ± 298.1, P = 0.78).

Table 5 compares TTE PH parameters in those with a low TTE probability and RHC PH to those with no RHC PH. None of the BSE/ESC TTE PH probability parameters were significantly different between those with PH and without. RVFWLS however was significantly lower in those with PH (P < 0.02). In addition to RVFWLS, RVFAC was lower in those with PH (p = 0.05) and significantly lower when excluding those with LHD (P < 0.01). Three with PH had an RV wall thickness > 5 mm compared to none without PH (P < 0.03). Those with PH and low TTE PH probability had significantly higher biplane LA volumes (67.9 ± 39.4 mL vs 49.4 ± 18.6 mL) (P < 0.05) and E/e′ (9.3 ± 3.5 vs 6.5 ± 2.3) (P < 0.05) reflecting the higher proportion of PH due to LHD. There was also a trend towards higher IVRT values in those with PH (NS).

**Table 5:** Summary of PH Echocardiographic parameters in those with low TTE probability and with PH or no PH (N = 78)

Echocardiographic parameter	No Pulmonary Hypertension (N = 51)	Pulmonary hypertension (N = 28)	Statistical significance
Average Peak TRV (m/s)	2.34 ± 0.31	2.36 ± 0.43	NS
Average RV/LV basal diameter ratio	0.72 ± 0.12	0.77 ± 0.14	NS
End-Diastolic Eccentricity index	0.91 ± 0.12	0.91 ± 0.13	NS
End-Systolic Eccentricity index	0.97 ± 0.12	1.0 ± 0.17	NS
Early Peak Diastolic PRV (m/s)	1.58 ± 0.31	1.7 ± 0.44	NS
Average MPA diameter (mm)	19.4 ± 4	20.6 ± 7.2	NS
RVOT acceleration time (ms)	114.6 ± 31.6	102.6 ± 29.8	NS
Frequency of MSN notching (%)	6	7	NS
Frequency of IVC diameter > 21 mm with < 50% Respiratory collapse	0	4	NS
RAA (cm^2^)	14 ± 4	14.6 ± 6.5	NS
RVFWLS (–%)	− 23.8 ± 3.9	− 20.6 ± 4.1	P < 0.02
RV TDI IVRT (ms)	71.5 ± 23.9	80.3 ± 22	NS
RV TDI E’	10.3 ± 3.1	10.1 ± 3.7	NS
RV TDI E’:A’ ratio	0.83 ± 0.38	0.8 ± 0.39	NS
RV wall thickness > 5 mm (n = 14)	0/9	3/5 (60)	P < 0.03
RAAI (cm^2^/m^2^)	7.1 ± 2.1	7.65 ± 3	NS
RVD-1	3.38 ± 0.64	3.59 ± 0.63	NS
RVEDAI (cm^2^/m^2^)	9.34 ± 2.1	8.41 ± 3.45	NS
RVFAC (%)	38.6 ± 9.7	32.7 ± 11.1	P = 0.05
TAPSE (cm)	2.17 ± 0.45	2.08 ± 0.4	NS
RV S’ (cm/s)	14.04 ± 3.28	13.08 ± 3.25	NS
Pericardial effusion present (%)	4	8	NS

*TTE* transthoracic echocardiography, *PH* pulmonary hypertension, *TRV* tricuspid regurgitant velocity, *RV* right ventricle, *LV* left ventricle, *PRV* pulmonary regurgitant velocity, *MPA* main pulmonary artery, *RVOT* right ventricular outflow tract, *MSN* mid-systolic notching, *IVC* inferior vena cava, *RAA* right atrial area, *RVFWLS* right ventricular free wall longitudinal strain, *TDI* tissue doppler imaging, *IVRT* isovolumetric relaxation time, *RAAI* right atrial area indexed, *RVFAC* right ventricular fractional area of change, *RVEDAi* right ventricular end-diastolic area indexed, *TAPSE* tricuspid annular plane excursion

### IMPULSE algorithm

The three markers in the IMPULSE algorithm (Fig. 2) were developed because they either demonstrated a significant difference between those with/without PH or suggested a trend towards abnormality in those with PH. ROC curve analysis of markers including RVFWLS, TDI IVRT and RVFAC also demonstrated comparable diagnostic accuracy when compared to conventional PH TTE markers.

TTE PH probabilities in those with low or intermediate probability were re-evaluated using the IMPULSE algorithm which included RVFWLS, RVFAC and IVRT as additional markers of PH probability. Those who had either an RVFWLS < − 23% or reduced RVFAC (< 35% female or < 30% male) in combination with an IVRT TDI > 73 ms were deemed IMPULSE positive and therefore hypothesised to be more likely to have PH than those IMPULSE negative. Cut-off values are reflective of current BSE guidance [18].

The IMPULSE algorithm however could only be retrospectively used in 52% of those with low or intermediate TTE probability. The number of false negatives in those who had low BSE/ESC TTE PH probability (N = 28) was reduced by 29% (N = 8). Of these, 4 had pre-capillary PH (N = 2 WHO group 4, N = 2 WHO group 1). The remaining 4 had PH secondary to LHD.

In those with intermediate TTE probability and PH, IMPULSE correctly identified 85% (N = 11) of those with pre-capillary PH. IMPULSE was achievable in N = 13 (43%) of those with intermediate TTE probability and pre-capillary PH (N = 30). IMPULSE was negative in all of the 5 patients with LHD (N = 5) where IMPULSE was measurable.

The IMPULSE algorithm had excellent specificity and positive predictive value in those with a low (93% / 80%, respectively) or intermediate (82% / 86%, respectively) BSE/ESC TTE PH probability (Table 6). This compares to a positive predictive value of 74% with a BSE/ESC intermediate TTE probability only.

**Table 6:** Calculated sensitivity, specificity, negative predictive value and positive predictive value of using the IMPULSE algorithm in those with low or intermediate TTE probability

	Low TTE probability (%)	Intermediate TTE probability (5)
Sensitivity	44	46
Specificity	93	82
NPV	74	39
PPV	80	86

## Discussion

### BSE/ESC TTE PH probability

PH is a progressive and life-limiting disease. Early recognition and treatment are key in determining long-term prognosis [1]. Optimal screening methods for PH involve early identification of risk factors as well as a multi-modality approach with TTE often being the first line investigation [2]. Our findings support the use of a multi-parameter TTE approach to the screening of suspected PH. In our population of patients referred to a PH centre ESC/BSE TTE probability algorithm [2, 4] provides high sensitivity but moderate specificity for identifying individuals at risk of PH. We have shown that whilst 96% of those with a high BSE/ESC TTE PH probability had RHC PH, 22% of those with an intermediate BSE/ESC TTE PH probability did not have RHC PH. Strikingly, 36% of patients with a low BSE/ESC TTE PH probability had PH. These patients typically displayed milder forms of PH haemodynamics at RHC and therefore are less likely to have positive BSE/ESC TTE PH echocardiographic markers. No clinical markers were able to distinguish those who had pre-capillary PH in this group. Only those with LHD associated PH and low TTE probability had significantly higher NT-proBNP compared to those without PH. In addition, none of the TTE parameters used in the BSE/ESC TTE PH probability algorithm were significantly different between those with or without PH. Therefore, additional echocardiographic techniques are needed to identify patients earlier in the ‘PH cascade’.

Obtaining an accurate peak TRV measurement is crucial to assessing the TTE PH probability. Peak TRV’s were unmeasurable in 8% of PH patients; 53% of whom had low TTE probability. However, even in those with a measurable TRV just under half of those with a peak TRV ≤ 2.8 m/s had PH. The most likely explanation is measurement of suboptimal Doppler signals [18], adding further evidence to suggest a cut-off TRV alone would be unreliable in detecting PH [2, 4]. Accurate measurement of the peak TRV can however be improved with the use of agitated saline [4].

The MPA diameter was frequently unmeasurable due to suboptimal imaging. There is a lack of evidence demonstrating that MPA dilatation is a sensitive marker at detecting PH [18]. Our data suggests MPA dilatation shows poor sensitivity but high specificity in detecting PH. It could be concluded that more sensitive markers of PH could replace MPA dilation in the TTE algorithm. Retrospective measurement of the PRV_BD_ was frequently hampered by poor PR Doppler optimisation.

The remaining TTE parameters were frequently measurable but were often only abnormal in those with higher mPAP at RHC. These markers often provided good specificity but low sensitivity for detecting milder forms of PH. This is perhaps explained by the fact that RV hypertrophy (RVH) precedes dilatation during progressive RV pressure overload in PH [18, 19]. Right atrial area indexed was significantly higher in those with PH further increasing specificity however at the expense of sensitivity. These markers were highly specific for PH but were unable to identify those earlier in the disease process.

We have shown that the existing cut-offs for the currently used BSE/ESC echocardiographic parameters to assess the probability of PH are similar to the optimal values in our real-world cohort. This reinforces our findings that additional echocardiographic markers to assess the probability of PH are needed in those with milder forms of PH which current echocardiographic measures may not be sufficient to detect. We have found that using a combination of RVFWLS, RVFAC and RV IVRT may help to identify those with milder forms of PH. Theses markers represent sensitive indices of RV longitudinal, radial and diastolic function parameters.

#### RVFAC

Measuring RVFAC is recommended when evaluating RV function in PH [18]. Gender specific cut-off values of normality are recommended [20]. We found RVFAC to be significantly lower in those with low BSE/ESC TTE PH probability and pre-capillary PH. It was also significantly lower in those with intermediate BSE/ESC TTE PH probability and PH compared to those without PH. It is thought that radial function is more predictive of global RV function in states of increased afterload such as PH [21]. In addition, there is an increased contribution of circumferential and radial shortening to RV function in PH patients due to RVH [19]. Conventional measures of RV longitudinal function including TAPSE and RV S’ therefore may not reflect global RV dysfunction in PH, especially in milder forms of PH. RVFAC is a simple method of assessing RV systolic function that has been shown to correlate with prognosis and response to treatment in PH patients [22, 23]. However, accurate measurements can be limited by poor endocardial definition and off-axis imaging [24]. We found that RVFAC was retrospectively measurable in 65% of those with low TTE probability. However, with an increased focus on obtaining suitable imaging to accurately measure RVFAC the proportion in whom this measurement is available in would likely increase.

#### RVFWLS

Whilst RVFAC incorporates RV radial assessment, RVFWLS is a sensitive measure of longitudinal function. LV GLS has been routinely utilised to identify early markers of LV dysfunction [25, 26]. Previous studies have shown RVFWLS is reduced in those with PH compared to controls and also adds prognostic value [13, 14]. RVFWLS is also closely correlated to RV ejection fraction by CMR [27]. However, to our knowledge no studies have evaluated RVFWLS in screening for PH. We have demonstrated that RVFWLS is able to help distinguish those with PH from those without in a cohort of low BSE/ESC TTE PH probability patients. RVFWLS was also lower in those with PH and an intermediate TTE probability although did not reach statistical significance. This may have been due to low number of patients with no PH and measurable RVFWLS in this group (N = 12). It is important to note however that no one TTE marker in isolation can identify PH [18]. In addition, baseline RVFWLS can provide prognostic information. RVFWLS is believed to be less susceptible to the assumptions and limitations of conventional parameters of RV function including angle dependency [14, 28]. Including RVFWLS in those with a low or intermediate TTE probability may help identify those with PH.

There are limitations associated with RVFWLS. It relies on good endocardial definition and therefore isn’t achievable in every patient. Retrospective measurements were made in just over half of those with low or intermediate TTE probability. However, prospective measurements are likely to be more feasible [29, 30]. Previous literature has suggested good reproducibility in RVFWLS measurements [14, 30]. In addition, there is no definitive cut-off value of normality across vendor platforms for RVFWLS. A provisional cut-off of -23% has been suggested which was the level that provided optimal sensitivity and specificity for detecting PH in our cohort [17, 31]. The routine adoption of RVFWLS has been previously limited by inter-vendor variability [17]. Guidance on the standardisation of RVFWLS measurement has been published including the use of the RV focused A4C view and dedicated RVFWLS analysis software [17]^.^ [31].

### RV diastolic dysfunction

We hypothesised that markers of RV diastolic dysfunction may help identify those with PH and low or intermediate TTE PH probability. To our knowledge no previous studies have assessed the use of RV diastolic markers in screening for PH. RV E′ and RV TDI E′/A′ ratios were significantly lower in those with PH. TDI IVRT was significantly prolonged in those with PH, displaying good sensitivity but moderate specificity in detecting PH. There was a trend for higher average IVRT values in those with intermediate and low BSE/ESC TTE PH probability and PH compared to those without PH. An increased IVRT reflects poor myocardial relaxation [18]. Studies have demonstrated a reduction in IVRT durations in PH patients using Sildenafil therapy [17]. A comprehensive assessment of RV diastolic dysfunction was limited given PW Doppler interrogation of tricuspid inflow was used infrequently as it is not part of the standard minimum dataset [32].

### IMPULSE algorithm

Our findings suggest a combination of additional TTE makers such as RVFWLS, RVFAC and IVRT in those with low or intermediate BSE/ESC determined probability may increase the detection of PH in these groups. Using the IMPULSE algorithm reduced the number of false negatives with pre-capillary PH and low TTE probability by 44%. It also had a superior PPV compared to intermediate BSE/ESC TTE PH probability alone. The IMPULSE algorithm however could only be retrospectively used in 52% of those with low or intermediate TTE probability. IMPULSE was often unmeasurable due to no RV TDI imaging being stored or endocardial definition being suboptimal for RVWLS or RVFAC measurements. Prospective measurements however are likely more feasible. Further prospective research is needed to confirm the improved sensitivity of the IMPUSLE algorithm amongst patients with low/intermediate probability PH using existing tools.

### Limitations

Our findings represent those of a single centred retrospective study in a cohort of patients referred to a shared care national PH service, thus the number of patients without PH is low compared to screening populations. Therefore, the clinical suspicion of PH is increased. RVFAC and RVFWLS were not measurable in all those with a low or intermediate TTE probability and PH. Retrospective RVFWLS measurements were not performed using dedicated RVFWLS software which may have influenced the accuracy of some measurements [18]. Differences in RVFWLS values may exist between vendors however all measurements in this study used the same vendor software. 2D RVFWLS measurements in our study did not consider the complex 3D geometry of the RV with future studies including 3D RVFWLS needed. Typically, retrospective measurements of PRV_BD_ could not be performed due to poor Doppler optimisation of the PR jet. The TTE parameters assessed were limited by those that could be retrospectively measured from the stored data and therefore did not evaluate all possible right heart indices such as hepatic venous flow or trans-tricuspid Doppler for example.

TTE was not performed simultaneously with RHC. The median interval from TTE to RHC was 24 days and therefore comparisons between echocardiographic and invasive measures may have been influenced by varying haemodynamic loading conditions.

## Conclusion

Current international guidelines for the echocardiographic probability of PH may not be sensitive enough to detect patients with milder haemodynamic forms of PH. Our findings suggest a combination of additional TTE makers assessing RV radial, longitudinal and diastolic function are helpful in identifying those with milder forms of PH, particularly in those who have a low BSE/ ESC TTE PH probability. Incorporating RVFWLS, RVFAC and IVRT in those with low or intermediate BSE/ESC determined probability may reduce the number of false negatives, improve specificity and positive predictive value when compared to current guidelines.

## Supplementary Information

**Additional file 1: Figure S1a:** Comparison of average RV free wall longitudinal strain (RVFWLS) in those with a high, intermediate or low TTE probability as well as those without PH. Kruskal–Wallis analysis demonstrated RVFWLS was significantly different relative to TTE PH probability group; H = 51.73 (3) (P < 0.001). Pairwise analysis with adjusted P-values demonstrated that RVFWLS values were significantly lower in those with high TTE probability compared to those with low intermediate TTE probability and those without PH (all P < 0.001). There was no significant difference in strain values between low and intermediate probabilities (p > 0.05). **Figure S1b:** Comparison of RVFWLS values relative to WHO classification of PH. Kruskal–Wallis analysis showed no statistically significant difference in RVFWLS values relative to WHO classification in either group (p > 0.05). RVFWLS values however were significantly higher in those without PH compared to all WHO classifications of PH, H = 31.12 (5) (all P < 0.05). **Figure S2a:** Comparison of frequencies of PH in those with a low TTE probability (n = 78) (left) and average RVFWLS in those Low TTE probability with PH (= 23) and without (n = 27) (right). RVFWLS was significantly lower in those with PH and low TTE probability (P < 0.02). **Figure S2b:** Comparison of frequencies of PH in those with an intermediate (n = 36) echocardiographic probability (left) and average RVFWLS in those with intermediate TTE probability in those with PH (n = 24) and without (n = 12) (right). **Figure S3:** ROC curve analysis of RV TDI IVRT (ms) N = 164. AUC 0.72 (95% CI 0.62–0.82). Using an IVRT cut-off value of > 73 ms demonstrated sensitivity and specificity of 81% and 62%, respectively for detecting PH. **Figure S4a:** Comparison of RV TDI e’ (cm/s) relative to WHO classifications of PH. Kruskal–Wallis analysis demonstrated RV TDI e’ values were different across WHO classifications of PH; H = 31.7(5) P < 0.001. Pairwise analysis with adjusted p-values showed that RV e’ values were significantly lower in those with CTEPH (P < 0.01) and PAH (P < 0.02) compared to those with PH due to LHD. RV e’ values were not significantly different between those with no PH and LHD or multifactorial PH (p > 0.05). **Figure S4b:** Comparison of RV TDI E’:A’ ratios relative to WHO classifications of PH. Kruskal–Wallis analysis demonstrated E/A ratios were significantly different across WHO classifications of PH H = 33.1(5), P < 0.001). RV TDI E’:A’ ratios were significantly lower in those with PAH (P < 0.02), CTEPH (P < 0.01) and lung disease (P < 0.02) compared to those with LHD. E/A ratios were not significantly different between those without PH and those with LHD or multifactorial PH (p > 0.05). **Figure S4c:** Comparison of RV TDI IVRT (ms) relative to WHO classifications of PH. Kruskal–Wallis analysis demonstrated IVRT values were not statistically significantly different across WHO classifications of PH. However, IVRT values were significantly higher in those with PAH (P < 0.001) and CTEPH (P < 0.01) compared to those without PH (H = 24.3(5) P < 0.001). **Figure S5a:** Comparison of average RV TDI IVRT values (ms) in those with low TTE probability with PH (N = 17) and without (N = 28). RV TDI IVRT (right ventricular tissue Doppler imaging isovolumetric relaxation times). **Figure S5b:** Comparison of average RV TDI IVRT values (ms) in those with intermediate TTE probability in those with PH (N = 27) and without N = 13). RV TDI IVRT (right ventricular tissue Doppler imaging isovolumetric relaxation times). **Table S1:** Population characteristics (N = 310). **Table S2:** Comparison of echocardiographic markers of RV diastolic dysfunction in those with and without PH. **Table S3:** Receiver operating characteristic derived cut-off values for the TTE PH parameters. **Table S4:** Re-audit of the TTE PH probabilities using ROC curved defined cut-off values compared to BSE/ESC algorithm.

## Data Availability

The data that support the findings of this study are available on reasonable request from the corresponding author DXA.

## Abbreviations

A4C: Apical four chamber
AFI: Automated function imaging
BSA: Body surface area
BSE: British Society of Echocardiography
CAD: Coronary artery disease
CI: Confidence interval
CMR: Cardiac magnetic resonance imaging
COPD: Chronic obstructive pulmonary disease
CTD: Connective tissue disease
CTEPH: Chronic thromboembolic pulmonary hypertension
CW: Continuous wave
ESC: European Society of Cardiology
GE: General electric
GLS: Global longitudinal strain
IMPULSE: Improving pulmonary hypertension screening by echocardiography
IVC: Inferior vena cava
IVRT: Isovolumetric relaxation time
IQR: Inter quartile range
LA: Left atrium
LHD: Left heart disease
LV: Left ventricular
MPA: Main pulmonary artery
mPAP: Mean pulmonary artery pressure
MSN: Mid systolic notching
NYHA: New York Heart Association
NT pro BNP: N-terminal pro hormone of brain natriuretic peptide
PAH: Pulmonary arterial hypertension
PASP: Pulmonary artery systolic pressure
PCWP: Pulmonary capillary wedge pressure
PH: Pulmonary hypertension
PR: Pulmonary regurgitation
PRV_BD_: Pulmonary regurgitation velocity at beginning of diastole
PVR: Pulmonary vascular resistance
PW: Pulsed wave
RAA: Right atrial area
RAAI: Right atrial area indexed
R&D: Research and development
RAP: Right atrial pressure
RHC: Right heart catheterisation
ROC: Receiver operating characteristic
RV: Right ventricle
RVFAC: Right ventricular fractional area of change
RVFWLS: Right ventricular free wall longitudinal strain
RVH: Right ventricular hypertrophy
RVOT: Right ventricular outflow tract
RVSP: Right ventricular systolic pressure
SPSS: Statistical package for social sciences
TAPSE: Tricuspid annular peak systolic excursion
TDI: Tissue Doppler imaging
TRV: Tricuspid regurgitant velocity
TTE: Transthoracic echocardiography
UK: United Kingdom
WHO: World Health Organisation
WU: Woods units
6MWT: Six-minute walk test
