# Supplementary material for: IMproving PULmonary hypertension Screening by Echocardiography: IMPULSE
Source: Echo Res Pract. 2022 Oct 19;9:9. doi: 10.1186/s44156-022-00010-9 (PMC9580132; doi:10.1186/s44156-022-00010-9)
Supplement: Supplementary file 1 — Additional file 1: Figure S1a: Comparison of average RV free wall longitudinal strain (RVFWLS) in those with a high, intermediate or low TTE probability as well as those without PH. Kruskal–Wallis analysis demonstrated RVFWLS was significantly different relative to TTE PH probability group; H = 51.73 (3) (P < 0.001). Pairwise analysis with adjusted P-values demonstrated that RVFWLS values were significantly lower in those with high TTE probability compared to those with low intermediate TTE probability and those without PH (all P < 0.001). There was no significant difference in strain values between low and intermediate probabilities (p > 0.05). Figure S1b: Comparison of RVFWLS values relative to WHO classification of PH. Kruskal–Wallis analysis showed no statistically significant difference in RVFWLS values relative to WHO classification in either group (p > 0.05). RVFWLS values however were significantly higher in those without PH compared to all WHO classifications of PH, H = 31.12 (5) (all P < 0.05). Figure S2a: Comparison of frequencies of PH in those with a low TTE probability (n = 78) (left) and average RVFWLS in those Low TTE probability with PH (= 23) and without (n = 27) (right). RVFWLS was significantly lower in those with PH and low TTE probability (P < 0.02). Figure S2b: Comparison of frequencies of PH in those with an intermediate (n = 36) echocardiographic probability (left) and average RVFWLS in those with intermediate TTE probability in those with PH (n = 24) and without (n = 12) (right). Figure S3: ROC curve analysis of RV TDI IVRT (ms) N = 164. AUC 0.72 (95% CI 0.62–0.82). Using an IVRT cut-off value of > 73 ms demonstrated sensitivity and specificity of 81% and 62%, respectively for detecting PH. Figure S4a: Comparison of RV TDI e’ (cm/s) relative to WHO classifications of PH. Kruskal–Wallis analysis demonstrated RV TDI e’ values were different across WHO classifications of PH; H = 31.7(5) P < 0.001. Pairwise analysis with adjusted p-values showe [file 44156_2022_10_MOESM1_ESM.docx]

**Additional file 1**

A

B

**Figure S1a:** *Comparison of average RV free wall longitudinal strain (RVFWLS) in those with a high, intermediate or low TTE probability as well as those without PH. Kruskal-Wallis analysis demonstrated RVFWLS was significantly different relative to TTE PH probability group; H=51.73 (3) (P<0.001). Pairwise analysis with adjusted P-values demonstrated that RVFWLS values were significantly lower in those with high TTE probability compared to those with low intermediate TTE probability and those without PH (all P<0.001). There was no significant difference in strain values between low and intermediate probabilities (p>0.05).* **Figure S1b:** *Comparison of RVFWLS values relative to WHO classification of PH. Kruskal-Wallis analysis showed no statistically significant difference in* RVFWLS *values relative to WHO classification in either group (p>0.05). RVFWLS values however were significantly higher in those without PH compared to all WHO classifications of PH, H=31.12 (5) (all P<0.05).*

A

B


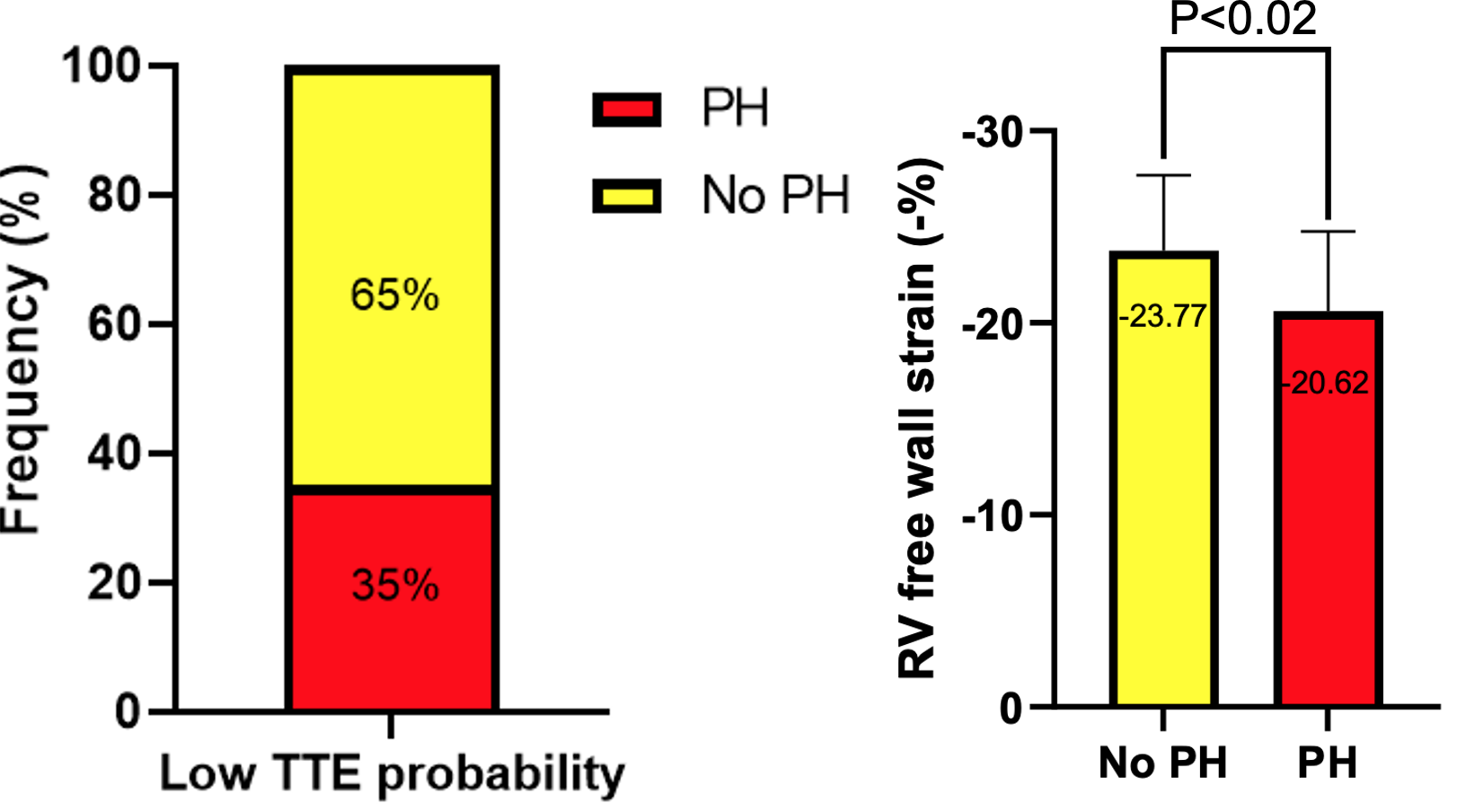

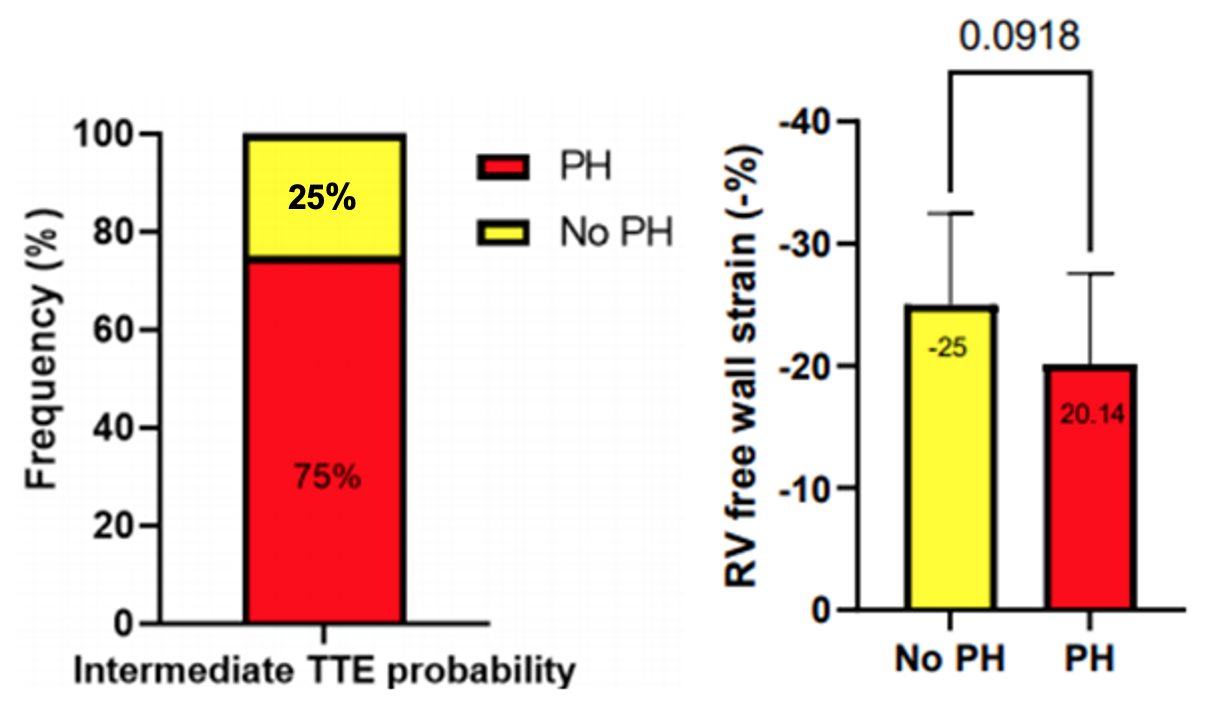


**Figure S2a:** *Comparison of frequencies of PH in those with a low TTE probability (n=78) (left) and average RVFWLS in those Low TTE probability with PH (=23) and without (n=27) (right). RVFWLS was significantly lower in those with PH and low TTE probability (P<0.02).* **Figure S2b:** *Comparison of frequencies of PH in those with an intermediate (n=36) echocardiographic probability (left) and average RVFWLS in those with intermediate TTE probability in those with PH (n=24) and without (n=12) (right).*

*
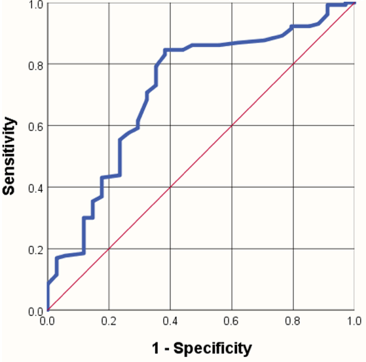
*

***Figure S3:*** *ROC curve analysis of RV TDI IVRT (ms) N=164. AUC 0.72 (95% CI 0.62-0.82). Using an IVRT cut-off value of >73ms demonstrated sensitivity and specificity of 81% and 62%, respectively for detecting PH,*

B

A


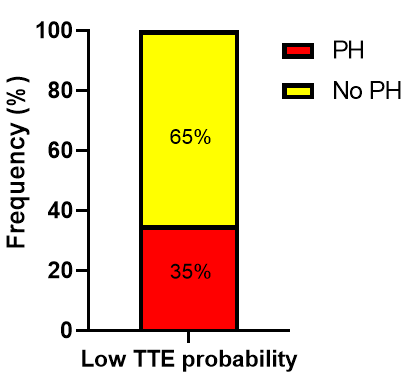

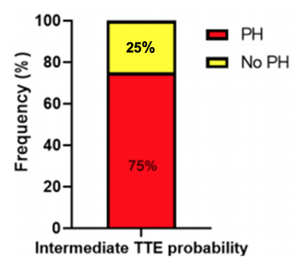


C

A

B

***Figure S5a:*** *Comparison of average RV TDI IVRT values (ms) in those with low TTE probability with PH (N=17) and without (N=28). RV TDI IVRT (right ventricular tissue Doppler imaging isovolumetric relaxation times).* **Figure S5b:** *Comparison of average RV TDI IVRT values (ms) in those with intermediate TTE probability in those with PH (N=27) and without N=13). RV TDI IVRT (right ventricular tissue Doppler imaging isovolumetric relaxation times).*

**Figure S4a:** *Comparison of RV TDI e’ (cm/s) relative to WHO classifications of PH. Kruskal-Wallis analysis demonstrated RV TDI e’ values were different across WHO classifications of PH; H=31.7(5) P<0.001. Pairwise analysis with adjusted p-values showed that RV e’ values were significantly lower in those with CTEPH (P<0.01) and PAH (P<0.02) compared to those with PH due to LHD. RV e’ values were not significantly different between those with no PH and LHD or multifactorial PH (p>0.05).* ***Figure S4b:*** *Comparison of RV TDI E’:A’ ratios relative to WHO classifications of PH. Kruskal-Wallis analysis demonstrated E/A ratios were significantly different across WHO classifications of PH H=33.1(5), P<0.001). RV TDI E’:A’ ratios were significantly lower in those with PAH (P<0.02), CTEPH (P<0.01) and lung disease (P<0.02) compared to those with LHD. E/A ratios were not significantly different between those without PH and those with LHD or multifactorial PH (p>0.05).* ***Figure S4c:*** *Comparison of RV TDI IVRT (ms) relative to WHO classifications of PH. Kruskal-Wallis analysis demonstrated IVRT values were not statistically significantly different across WHO classifications of PH. However, IVRT values were significantly higher in those with PAH (P<0.001) and CTEPH (P<0.01) compared to those without PH (H=24.3(5) P<0.001).*

| Table S1. Population characteristics (N=310) | |
| --- | --- |
| Characteristics |  |
| Age (y) | 67±14 |
| Female (%) | 62 |
| BSA (m^2^) | 1.92±0.27 |
| Frequent comorbidities: |  |
| - *CAD* | 64 (24%) |
| - *Atrial fibrillation/flutter* | 55 (18%) |
| - *Systemic hypertension* | 101 (33%) |
| - *Hypercholesterolemia* | 7 (2%) |
| - *History of CVA* | 13 (4%) |
| - *Diabetic* | 29 (9%) |
| - *Smoking history* | 46 (15%) |
| - *Chronic kidney disease* | 30 (10%) |
| - *History of cancer* | 16 (5%) |
| - *History of pulmonary embolisms* | 72 (23%) |
| - *Chronic thromboembolic disease* | 8 (3%) |
| - *History of deep vein thrombosis* | 13 (4%) |
| - *Systemic sclerosis* | 34 (11%) |
| - *Scleroderma* | 13 (4%) |
| - *COPD* | 33 (11%) |
| - *Pulmonary fibrosis* | 10 (3%) |
| - *NSIP* | 9 (3%) |
| - *Obstructive sleep apnoea* | 9 (3%) |
| - *ILD* | 6 (12%) |

*BSA (Body Surface Area); CAD (coronary artery disease); CVA (cerebrovascular Accident); COPD (Chronic Obstructive Pulmonary Disease); ILD (interstitial lung disease); NSIP (Nonspecific Interstitial Pneumonia).*

| Table S2. Receiver operating characteristic derived cut-off values for the TTE PH parameters | | | |
| --- | --- | --- | --- |
| TTE parameter | ROC curve cut-off value | Sensitivity | Specificity |
| Lower peak TRV (m/s) | >2.8 | 83% | 73% |
| Upper peak TRV (m/s) | >3.2 | 72% | 94% |
| End systolic eccentricity index | >1.08 | 65% | 82% |
| End diastolic eccentricity index | >1.07 | 49% | 90% |
| RV/LV basal diameter ratio | >0.90 | 58% | 82% |
| RVOT acceleration time (ms) | <110 | 87% | 54% |
| MPA diameter (mm) | >21 | 60% | 59% |
| PRV_BD_ (m/s) | >2.1 | 70% | 94% |
| Right atrial area (cm^2^) | >16 | 65% | 71% |

*TTE (Transthoracic echocardiography); AUC (area under curve); CI (confidence interval); RVOT (right ventricular outflow tract); PRV_BD_ (pulmonary regurgitation velocity beginning of diastole); PH (pulmonary hypertension); TRV (tricuspid regurgitant velocity); RV (right ventricle) LV (left ventricle); PRV_BD_ (pulmonary regurgitant velocity at beginning of diastole); MPA (main pulmonary artery); RVOT (right ventricular outflow tract).*

| Table S3. Re-audit of the TTE PH probabilities using ROC curved defined cut-off values compared to BSE/ESC algorithm. | | | |
| --- | --- | --- | --- |
| TTE PH probability | ***Low*** | ***Intermediate*** | ***High*** |
| Using BSE/ESC cut-off values | | | |
| No PH (n=74) | 50 | 18 | 6 |
| PH (n=236) | 28 | 50 | 158 |
| BSE/ESC sensitivity and specificity | 88% and 68% | | |
| Using ROC curve cut-off values of study cohort |  | | |
| No PH (n=74) | 51 | 17 | 6 |
| PH (n=236) | 28 | 51 | 157 |
| New sensitivity and specificity | 88% and 69% | | |

*ESC (European Society of Cardiology); BSE (British Society of Echocardiography). PH (pulmonary hypertension).*

| Table S4. Comparison of echocardiographic markers of RV diastolic dysfunction in those with and without PH. | | | |
| --- | --- | --- | --- |
|  | Frequency (%) in those with PH (n=236) | Frequency (%) in those with without PH (n=74) | Statistical significance |
| Indexed Right Atrial Area >11cm^2^/m^2^ | 28% | 7% | P<0.001 |
| RV Wall Thickness >5mm | 13% | 0% | P<0.001 |
| IVC size and collapse (>21mm and <50%) | 17% | 3% | P<0.001 |
| RV TDI E’/A’ ratio <1 | 22% | 61% | P>0.05 |
| RV TDI IVRT >73ms | 69% | 21% | P<0.001 |
| RV TDI E’ <8 | 39% | 19% | P<0.01 |

*Data is displayed as a percentage frequency of those displaying features of RV diastolic dysfunction relative to the number of patients these echocardiographic parameters were measurable in. PH (pulmonary hypertension); RV (right ventricle); IVC (inferior vena cava); TDI (tissue Doppler imaging); IVRT (Isovolumetric relaxation time).*
